# Tuberculosis outbreak in a nursing home involving undocumented migrants and Israeli citizens

**DOI:** 10.1186/s13584-018-0219-y

**Published:** 2018-07-15

**Authors:** Z. Mor, N. Nuss, M. Savion, I. Nissan, M. Lidji, S. Maneshcu, H. Kaidar-Shwartz, Z. Amitai, E. Rorman, R. Sheffer

**Affiliations:** 1 Tel Aviv Department of Health, Ministry of health, 12 Ha’arba’a Street, 6473912 Tel Aviv, Israel; 2 Sackler School of Medicine, Tel Aviv University, Tel Aviv, Israel; 3 National Public Health Laboratory, Ministry of Health, Tel Aviv, Israel; 4 National Tuberculosis Reference Laboratory, Ministry of Health, Tel Aviv, Israel; 5 Tel Aviv Tuberculosis clinic, Tel Aviv, Israel

**Keywords:** Health care workers, Immigrants, Long term facility, Outbreak, Tuberculosis control

## Abstract

**Objectives:**

Israel has absorbed > 60,000 migrant from the horn of Africa (MHOA) since 2006. No cross-transmission of *Mycobacterium tuberculosis* from MOHA to Israeli citizens has yet been reported. This study describes the results of contact investigation and laboratory work-out of a unique mixed cluster which included both MOHA and Israeli citizens.

**Methods:**

Description of the results of epidemiological investigation including laboratory confirmation.

**Results:**

This unique *Mycobacterium tuberculosis* strain included 29 patients: 26 were MOHA and three citizens who immigrated to Israel from the former Soviet Union. This is the first mixed cluster described in Israel, which has not been represented in the SITVIT international database of genotyping markers. The transmission from non-citizens to citizens occurred in a nursing institution, when MOHA infected three other contacts- two of whom were retarded residents, one of them died. The index case was screened before employment, and was permitted to return to wok although his chest X-ray demonstrated radiological findings compatible with tuberculosis. Epidemiological links were found in other 12 MOHA members of the cluster.

**Conclusion:**

This report describes cross-transmission of *Mycobacterium tuberculosis* from non-citizens MOHA to Israeli citizens who were residents of a nursing home, which may be the first sign for an epidemiological shift. Although cross-ethnical transmission is still rare in Israel, medical settings should employ efficient infection control measures to protect both patients and staff from *Mycobacterium tuberculosis*.

## Background

During 2014 alone, 9.6 million individuals were diagnosed with tuberculosis, mostly in developed countries. Tuberculosis has been recently ranked as the leading cause of death from a single infectious agent, with a toll of 1.5 million people annually [1]. Due to the increasing trends of international migration, the proportion of foreign-born individuals of all tuberculosis cases is high in most industrialized countries.

Israel is a developed country of about 8 million citizens, with a gross domestic product of US$32,600 [2]. About 35% of the Israeli citizens were born elsewhere, and were naturalized upon arrival and are likely to assimilate in the Israeli society. In addition to these legal migrants, which are mostly Jews, Israel has recently become a destination of approximately 60,000 undocumented migrants from the horn of Africa who arrived between 2006 and 2012. More than half are residing in Tel Aviv [3], mostly in congregated housing, especially in the southern Tel Aviv neighborhoods. Due to the illegality of their stay, they are usually employed as laborers, mostly in undesirable professions.

The majority of tuberculosis patients in Israel are non-Israeli born, with an increasing proportion of undocumented infected migrants [4]. Most of these patients are undocumented migrants originated in the horn of Arica. They were screened for active tuberculosis at detention center upon arrival in Israel [5]. Those who were diagnosed with active disease were treated at detention. Those who are diagnosed in the community, after their release, are treated with a full course of anti-tuberculosis drugs regimen in the directly observed therapy, similarly to Israeli citizens [6]. These low-threshold clinics offer free medical care without concern of being reported to migration officials or threat of deportation.

Although transmission from migrants to local citizens has been previously described in other developed countries [7], no documented case of *Mycobacterial* transmission from the undocumented migrants originating in the horn of Africa to Israeli citizens has ever been reported until 2010 [8]. However, as these migrant are extending their stay in Israel and along with their greater assimilation in the local Israeli community, this trend may be overturned. This study describes a recent outbreak of tuberculosis in a nursing home involving migrants from the horn of Africa and Israeli citizens sharing an identical tuberculosis genotype.

## Methods

Tuberculosis case was defined as pulmonary or extra pulmonary-tuberculosis diagnosed by a pulmonologist specialized in tuberculosis by typical clinical symptoms, and additionally: had either direct sputum smear microscopy or culture positive with *M. tuberculosis*; and was prescribed a full course of anti-tuberculosis drugs for a period longer than three months due to tuberculosis-related symptoms, or due to abnormalities in chest radiography. All *Mycobacterium tuberculosis* isolates are characterized by the National Mycobacterium Reference Laboratory by routine DNA genotyping using 43 spacer spoligotyping (reverse line blot kit, Omicum, India) and 24 loci MIRU-VNTR (mycobacterial interspersed repetitive unit variable number tandem repeat) typing (in house multiplex PCR and amplicon-sizing by capillary electrophoresis) were done as previously described [9]. The epidemiological investigation included interview with the patients and their contacts, and extracting data from the medical files.

## Results of contact investigation

A 31 year old non-citizen male migrant from Eritrea was reported with tuberculosis in November 2012 (patient one). He arrived in Israel in 2010 and was employed at a long term facility for mentally retarded individuals in Tel Aviv since September 2011 as a caregiver. His complaints upon diagnosis included productive cough, fever, weakness and loss of appetite. His chest X ray (CXR) demonstrated a small cavitation in his left upper lobe and disseminated reticulonodular and peribronchial thickening. His sputum sample was positive and his culture showed drug sensitive *Mycobacterium tuberculosis*. A prior CXR, which was performed on June 2012 showed fibrotic changes combined with bronchial thickening in the left upper lobe. Nevertheless, he was permitted to return to his work.

An Epidemiological investigation, which included interviewing 68 residents and 32 staff member at the institution was shortly performed, followed by a two-step tuberculin skin test, which showed induration 5 mm or greater in 26 (38.2%) and two (6.2%), respectively. During the investigation in December 2012, one of the tenants, a 24 old male who was born in the former Soviet Union (FSU) and has been residing in the institution for three years, was diagnosed with pulmonary tuberculosis (patient two). As he was totally dependent due to a cerebral palsy, he was treated daily by his caregiver (patient one). His clinical symptoms started in October 2012, and included cough, fever and weight loss. His CXR showed left perihilar infiltration and large left pleural effusion with suspected infiltration. His sputum and gastric lavage were barren, while a plural fluid aspiration was positive for *Mycobacterium tuberculosis*, which was susceptible to all first line drugs and was diagnosed as extra pulmonary tuberculosis.

One year later, in December 2013, a 26 years old male Eritrean migrant, who arrived in Israel in 2010 and has been working in that institution since November 2011 was diagnosed with pulmonary tuberculosis (patient three). He complained of chest pain, hemoptysis, fever and weakness. His CXR demonstrated upper lobe infiltrates, and his sputum sample was positive. Culture results showed drug sensitive *Mycobacterium tuberculosis* which was identical in molecular typing to both patients one and two. Interestingly, he had tuberculin skin test showing 23 mm induration while he was tested during the epidemiological investigation following patient one’s diagnosis ten months earlier. He was recommended then preventive therapy with isoniazid for nine months. His adherence was partial and intermittent for only five months.

An additional 29 years old who was born in FSU and has resided in that institution since 2011, was admitted to the emergency room of a general hospital in December 2014 (patient four). She had an extensive medical record including cerebral palsy, with spastic tetraplegia, hydrocephalus, choreoathetosis, epilepsy and recurrent aspiration. This bed-ridden patient was previously examined during the epidemiological investigation following the former case (patient three), and her two-step tuberculin skin test performed on January 2013 was 0 mm. She was previously admitted to the hospital due to recurrent aspirations and consequent pneumonia. Her chest computerized tomography showed bilateral infiltrations with small cavitations. As she did not respond to polymixin E and a third generation cephalosporin, she had sputum sample examined, which was positive for *Mycobacterium tuberculosis*. Although she was treated with first line anti-tuberculosis drugs, she died two days later. Her culture showed drug sensitive *Mycobacterium tuberculosis* which was identical in molecular typing to all three former patients.

**Fig. 1:**
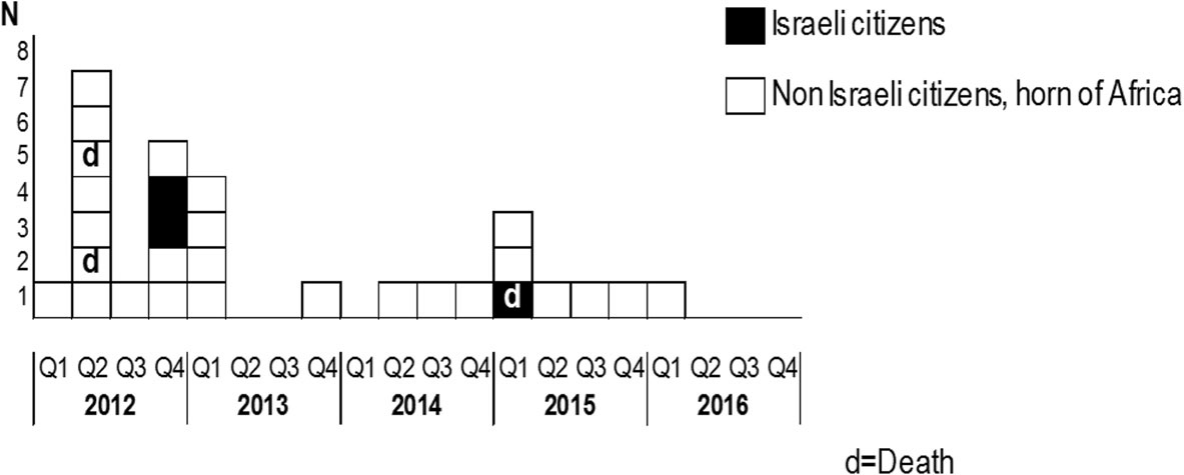
Histogram of newly diagnosed tuberculosis cases of spoligotyping octal pattern 777,737,377,720,771, 2012–2016. The molecular cluster of the four cases who were identified in the outbreak, which occurred in the medical institution, was similar to additional 25 tuberculosis patients, who were diagnosed in Israel between 2012 and 2016. Of the entire cluster, which includes 29 patients, 25 were born in Eritrea and one in Sudan - all were non-citizens migrants, while three patients were Israeli citizens who were born in the FSU

The molecular cluster of the four cases who were identified in the outbreak, which occurred in the medical institution, was similar to additional 25 tuberculosis patients, who were diagnosed in Israel between 2012 and 2016 (Table 1). Of the entire cluster, which includes 29 patients, 25 were born in Eritrea and one in Sudan - all were non-citizens migrants, while three patients were Israeli citizens who were born in the FSU (Fig. 1). Two of the Israeli citizens were tenants in the nursing home, and the third was also born in the FSU who lived one kilometer away from the institution in which the outbreak took place. However, no epidemiological link could be established. Of the non-citizens, 11 Eritreans (including patient one) were acquaints and played cards regularly in the same club in Tel Aviv. An additional Eritrean male patient was married to one of the women who played cards, and that woman was also in a close contact with another Eritrean man, who was infected. No epidemiological links were found between the other 11 non-citizen migrant patients who were also members of this cluster.

**Table 1:** Clinical and demographic characteristics of the 25 patients diagnosed with tuberculosis in this outbreak and the source case, Tel-Aviv district

patient	Age (years)	Sex	Contact	Country	TST 1st/2nd (mm)	Diagnosis date (month, year)	Smear/culture	symptoms	Chest X ray/Chest imaging	Site
1	31	M	Nursing home and card club	Eritrea	−/−	11/2012	Pos/Pos	Productive cough, fever, weakness, loss of appetite	Small cavitation in left upper lobe and disseminated reticulonodular and thickening	Pulmonary
2	24	M	Nursing home	FSU	14/−	12/2012	Neg/Pos (from pleura)	Fever, cough, weight loss	Large perihillar infiltrates with mediastinal shift, left pleural effusion and suspected infiltrations	Pleura
3	26	M	Nursing home	Eritrea	23/−	12/2013	Pos/Pos	Hemoptysis, weakness, fever, chest and back pain	Right upper lobe infiltration	Pulmonary
4	29	F	Nursing home	FSU	0/ 0	12/2014	Pos/Pos	Fever, dyspnea	Bilateral infiltrations with small left lower lobe shadowing and cavitation	Pulmonary
5	26	M	Card club	Eritrea	−/−	10/2012	Pos/Pos	Productive cough, weight loss, fever	Non-homogeneous bilateral diffuse shadowing with Right upper lobe cavitation	Pulmonary
6	72	M	Unknown	FSU	−/−	11/2012	Neg/Pos	Cough, pleuritic chest pain	Left lower lobe atelectasis	Pulmonary
7	23	M	Unknown	Eritrea	−/−	10/2012	Pos/Pos	Fever, abdominal pain, weakness, weight loss, cough	CT: Right lung multiple small nodular infiltration, (abdominal CT: ascites, omental soft tissue infiltration)	Pulmonary and abdominal
8	39	M	Card club	Eritrea	−/−	02/2012	Pos/Pos	Productive cough, weakness, weight loss,	Right upper lobe shadowing, small fibrotic changes at left upper lobe	Pulmonary
9	29	M	Unknown	Eritrea	−/−	04/2012	Pos/Pos	Dyspnea, weakness, productive cough	Bilateral infiltration and right large pleural effusion	Pulmonary
10	42	M	Unknown	Eritrea	−/−	04/2012	Pos/Pos	Fever. Productive cough, chest pain	Right upper lobe shadowing	Pulmonary
11	28	M	Married, friends	Eritrea	−/−	05/2012	Pos/Pos	Cough, weight loss, Weakness	Large Left upper lobe cavitation, multiple pulmonary foci, hematogenic spread	Miliary
12	21	M	Unknown	Eritrea	−/−	05/2012	Neg/Pos	Fever, cognitive decline, stupor	Diffuse perihilar shadowing	Miliary
13	20	M	Card Club	Eritrea	−/−	05/2012	Neg/Pos	Productive cough, white sputum	Left lower lobe shadowing	pulmonary
14	29	M	Card Club; friends	Eritrea	12 mm/−	03/2013	Pos/Pos	Weight loss, night sweats, productive cough	Right lower lobe-non homogeneous shadowing, small pleural effusion	Pulmonary
15	33	M	Unknown	Sudan	−/−	02/2013	Pos/Pos	shoulder pain, cough, fever, anorexia	Enhanced pulmonary pattern in left inferior lobe	Pulmonary and vertebral
16	35	M	Unknown	Eritrea	−/−	02/2013	Neg/Pos	Systematic symptoms	Left upper lung shadowing	Pulmonary
17	29	F	Unknown	Eritrea	−/−	02/2013	Neg/Pos	Chest pain, cough, chills, dyspnea	Right lower lobe shadowing with pleural effusion	Pleura
18	28	M	Unknown	Eritrea	−/−	06/2012	Neg/Pos	Fever, productive cough, weight loss, night sweats, anorexia, abdominal pain, emesis	Right hilar widening, lymphadenopathy	Pulmonary, lymph and thorax
19	30	M	Card club	Eritrea	−/−	03/2015	Pos/Pos	Fever, dyspnea, chest pain, cough	Wide consolidation, air broncho gram left lung	pulmonary
20	27	M	Card club	Eritrea	−/−	03/2015	Neg/Pos	Weight loss, chest pain	Normal chest x-ray	Vertebral
21	30	M	Card club	Eritrea	−/−	03/2015	Neg/pos	Fever, cough, dyspnea	Right pneumothorax, mediastinal shift, left perihilar shadowing	Pulmonary
22	40	M	Unknown	Eritrea	−/−	12/2015	Pos/Pos	Cough, anorexia, weight loss	Left upper lobe shadowing and infiltration	Pulmonary, abdominal, genito-urinary and lymph
23	28	M	Card club	Eritrea	−/−	05/2014	Neg/Pos	Cough	Bilateral infiltration, suspected cavitation	Pulmonary
24	26	F	Card Club, married	Eritrea	−/−	05/2012	Neg/Pos	Weakness, loss of appetite	Left pleural effusion, right hydropneumothorax	Pulmonary and pleura
25	23	M	Card club	Eritrea	−/−	04/2015	Neg/Pos	Chest pain, hemoptysis, weight loss, anorexia	Right upper lobe- consolidation with cavitation	Pulmonary
26	31	M	Unknown	Eritrea	−/−	09/2012	Pos/Pos	Cough, weight loss, weakness	Disseminated right middle lobe, left upper lobe infiltration, right middle lobe cavitation	Pulmonary

*F* Female, *FSU* Former Soviet Union, *M* Male, *Neg* negative, *Pos* Positive, *TST* Tuberculin screening test

Molecular typing of the *M. tuberculosis* isolates from these patients demonstrated identical 24 MIRU-VNTR and 43 spacer spoligotyping. Interestingly, these isolates characterized by a unique spoligotyping octal pattern 777,737,377,720,771, (24 MIRU-VNTR: 225313153523323222434572) not presented in the SITVIT international database.

## Discussion

This study describes the first evidence of *Mycobacterial* transmission which showed cross-transmission from non-citizens migrants to Israeli citizens, in a country in which tuberculosis epidemiology is constantly monitored by epidemiological investigations with molecular linkage followed each reported case.

Migrants who originate in developed countries, which are also characterized by high TB-prevalence, are at higher risk of developing TB. These migrants have the potential to expose the local population in the host country to *Mycobacterium tuberculosis*. However, no transmission has been demonstrated from non-citizen migrant to Israeli citizens until this study, and only limited transmission was observed from other Jewish citizens migrants to Israeli citizens who were locally born. Similar findings regarding the limited cross-ethnical transmission in Israel were demonstrated in a research showing minimal transmissions between Jews and Arabs, as they usually do not share households [10].

Most of the non-citizens migrants in Israel are living in poor and separated neighborhoods than the Israeli citizens, and very rarely do they share the same household with other Israeli citizens. Most of *Mycoabterial* transmission are therefore reported within the migrant community [11], while sparing the citizens of the hosting country, with whom they have limited social-links [12–14]. This in contrast with the xenophobic rhetoric fueled by some politicians and social leaders against migrants alarming that foreigners may spread contagions diseases to the local population to support anti-migrant policies. The case presented in this study is an exception, as the intimate contact between a bed-ridden patient and his caregiver resemble that of a household. This case also highlights the value of prompt TB screening and care of caregivers originating in countries characterized by high TB-prevalence who are working in Israel.

Although CXR-based active TB screening is performed in Israel to all undocumented migrants at the detention center in the Israeli-Egyptian border, it may not capture migrants who have latent tuberculosis infection. Latent infection may reactivate to active disease once the migrants are in the community [5], especially during the first few years following arrival in the hosting country [10]. Previous studies have documented that the majority of *Mycobacterial* infections among migrants are acquired in their host countries, and most of the cases of active tuberculosis are a result of recent transmissions in the hosting country, mainly due to reactivation [15]. The caregiver (patient one in this study) performed CXR five months prior to his diagnosis in November 2012. The film then demonstrated bronchial thickening in the left upper lobe, but the caregiver was nevertheless permitted to return work and was not referred for TB clinic. Patient three in this study had positive tuberculin skin test induration, but his adherence to preventive therapy was intermittent and shorter than recommended. It may be that small medical institutions, such as the one presented in this study, do not have the resources to comply with the regulation for safeguarding staff’s screening and follow-up. This misconduct could have been avoided if the institution had nominated a medical practitioner to overlook at staff screening and medical condition of health care worker, as mandated by the Ministry of Health, especially of those who were born in countries which are characterized by high prevalence of tuberculosis.

As the migrants from the horn of Africa extend their stay in Israel, the dynamic of *Mycobacterial* transmission between migrants and locals may be changed. During their stay, they can integrate with the general Israeli community, while sharing workspaces or households with locals [11, 16]. This pattern of social mixing may allow *mycobacterial* transmission between migrants and Israeli citizens, as also been demonstrated in this report.

Migrants may have prior experience with tuberculosis in their home-countries, however, they may be unfamiliar with the importance of preventive treatment for latent tuberculosis infections, as it is not usually been treated in their home countries [17]. In order to encourage the migrants to refer to TB clinics and adhere to preventive treatment, they should have positive interaction with the medical staff at the patient-centered TB-clinics, while ensuring conducive social policies and protecting human rights and minimizing stigma and discrimination. The staff should provide culturally competent support to increase patients’ trust and to ensure the adherence to preventive treatment [18]. In order to encourage early diagnosis, migrants should also be informed about the location of the TB-clinics in Israel and that the access to TB treatment and care is free and unrelated to their legal status. This information can be distributed in designated general care clinics serving the migrants community [19].

All the isolates of this cluster were characterized by a unique spoligotyping octal pattern 777,737,377,720,771, which has not been presented in the SITVIT international database. Similar octal pattern was described in two previous publications from Ethiopia [20, 21]. The first publication focused on tuberculosis patients in Bahir Dar city and its surroundings in northwest Ethiopia and describe a single tuberculosis patient (of total of 168 analyzed by spoligotyping) with this unique octal, that incorrectly identify as lineage H3, SIT 3134. The second paper described three patients with identical spoligotype pattern among farmers in central Ethiopia. MIRU-VNTR data was not provided in those publications, nevertheless, it corroborates the epidemiological conclusions regarding the origin of this cluster in the horn of Africa.

## Conclusions

In summary, TB transmissions in other crowded settings in Israel were previously reported, such as hospital [22], boarding school [23], prison [24] and extended family [25]. This report describes a new cluster of *Mycobacterium tuberculosis* among migrants originating in the horn of Africa, which has been infiltrated to Israeli citizen in a nursing home. The chain of transmission was supported with proved epidemiological contact and laboratory linkage between four patients who worked or resided in a nursing home. This highlights the need to promote careful screening and follow-up of health care workers and migrants who are employed in health facilities. Nevertheless, their follow-up might be interrupted because of lack of personal information, shortage of resources and potential movements of migrants [7]. Finally, physicians should include the possibility of tuberculosis diagnosis in patients with respiratory symptoms who are residing in nursing care institutions.
